# Genotoxic and Cytotoxic Properties of Zinc Oxide Nanoparticles Phyto-Fabricated from the Obscure Morning Glory Plant *Ipomoea obscura* (L.) Ker Gawl

**DOI:** 10.3390/molecules26040891

**Published:** 2021-02-08

**Authors:** Mahadevamurthy Murali, Satish Anandan, Mohammad Azam Ansari, Mohammad A. Alzohairy, Mohammad N. Alomary, Sarah Mousa Maadi Asiri, Ahmad Almatroudi, M. C. Thriveni, Sudarshana Brijesh Singh, Hittanahallikoppal Gajendramurthy Gowtham, Mohammed Aiyaz, Chandrashekar Srinivasa, Asna Urooj, Kestur Nagaraj Amruthesh

**Affiliations:** 1 Applied Plant Pathology Laboratory, Department of Studies in Botany, University of Mysore, Manasagangotri, Mysore 570006, Karnataka, India; botany.murali@gmail.com; 2 Department of Studies in Food Science and Nutrition, University of Mysore, Manasagangotri, Mysore 570006, Karnataka, India; satishanandan84@gmail.com (S.A.); asnaurooj@foodsci.uni-mysore.ac.in (A.U.); 3 Department of Clinical Nutrition and Dietetics, Sri Devaraj Urs Academy of Higher Education and Research, Kolar 563101, Karnataka, India; 4 Department of Epidemic Disease Research, Institutes for Research and Medical Consultations (IRMC), Imam Abdulrahman Bin Faisal University, Dammam 31441, Saudi Arabia; maansari@iau.edu.sa; 5 Department of Medical Laboratories, College of Applied Medical Sciences, Qassim University, Qassim 51431, Saudi Arabia; dr.alzohairy@gmail.com; 6 National Center for Biotechnology, Life Science and Environmental Research Institute, King Abdulaziz City for Science and Technology, Riyadh P.O. Box 6086, Riyadh 11442, Saudi Arabia; malomary@kacst.edu.sa; 7 Department of Biophysics, Institute for Research and Medical Consultations (IRMC), Imam Abdulrahman Bin Faisal University, Dammam 31441, Saudi Arabia; smAsiri@iau.edu.sa; 8 Central Sericultural Germplasm Resources Centre, Central Silk Board, Ministry of Textiles, Thally Road, TVS Nagar, Hosur 635109, Tamil Nadu, India; thrivenimc@gmail.com; 9 Department of Studies in Biotechnology, University of Mysore, Manasagangotri, Mysore 570006, Karnataka, India; brijeshrajput.bt@gmail.com (S.B.S.); gajendramurthygowtham@gmail.com (H.G.G.); reachaiyaz@gmail.com (M.A.); 10 Department of Studies in Biotechnology, Davangere University, Davangere 577007, Karnataka, India; chandru.s@davangereuniversity.ac.in

**Keywords:** cytotoxicity, genotoxicity, cell cycle analysis by flow cytometry, ZnO-NPs, HT-29 cells, *Allium cepa*, *Ipomoea obscura*

## Abstract

The study was undertaken to investigate the antioxidant, genotoxic, and cytotoxic potentialities of phyto-fabricated zinc oxide nanoparticles (ZnO-NPs) from *Ipomoea obscura* (L.) Ker Gawl. aqueous leaf extract. The UV-visible spectral analysis of the ZnO-NPs showed an absorption peak at 304 nm with a bandgap energy of 3.54 eV, which are characteristics of zinc nanoparticles. Moreover, the particles were of nano-size (~24.26 nm) with 88.11% purity and were agglomerated as observed through Scanning Electron Microscopy (SEM). The phyto-fabricated ZnO-NPs offered radical scavenging activity (RSA) in a dose-dependent manner with an IC_50_ of 0.45 mg mL^−1^. In addition, the genotoxicity studies of ZnO-NPs carried out on onion root tips revealed that the particles were able to significantly inhibit the cell division at the mitotic stage with a mitotic index of 39.49%. Further, the cytotoxic studies on HT-29 cells showed that the phyto-fabricated ZnO-NPs could arrest the cell division as early as in the G0/G1 phase (with 92.14%) with 73.14% cells showing early apoptotic symptoms after 24 h of incubation. The results of the study affirm the ability of phyto-fabricated ZnO-NPs from aqueous leaf extract of *I. obscura* is beneficial in the cytotoxic application.

## 1. Introduction

Nanotechnology is a multidimensional discipline that has the potential to transform all the fields of science. Nanoparticles have gained importance due to their surface area to volume ratio and the behaviors that result from that [1,2]. In light of the environmental and biological risks due to the toxicity of used chemicals in synthetic metal–oxide nanoparticles, biologically synthesized nanoparticles have gained considerable importance as they are considered to be stable, eco-friendly, and also possess biological properties [3,4,5,6]. Different methods have been employed for the synthesis of nanoparticles, of which the hydrothermal method is one of the alternatives to the synthetic methods, as it is simple, cost-effective, eco-friendly, and less hazardous [7,8,9]. Zinc oxide nanoparticles (ZnO-NPs) have gained more attention compared to other metal–oxide nanoparticles because of their broader applications in scientific fields, including biological applications [8,10,11]. Apart from microorganisms, plant extracts have been used as reducing or capping agents as these play a pivotal and versatile role during the synthesis of nanoparticles, which are essential for their functions and applications in various fields [7,12,13,14]. 

It has been well documented in the literature that the chemical synthesis of nanoparticles yields toxic byproducts that are not eco-friendly and cost-effective, and researchers are concentrating on alternative methods [4,5,6]. Biosynthesis of nanoparticles using plant extract has gained interest due to its abundance of sources available in the environment [8,9]. Plants have a rich source of secondary metabolites present in them, which act as reducing/capping agents during the synthesis of nanoparticles [6,12,13]. In the recent past, ZnO-NPs have been synthesized from every part of the plant, such as leaves [8], stem [15], tuber [16], root [14], flower [7], fruit [17], and seed [18]. They have also shown to possess many biological properties such as antioxidant, antimicrobial, genotoxicity, cytotoxicity, etc., thereby indicating the efficacy toward their application in the field of pharmaceuticals [5,8,9,14,15]. The toxicity of chemically synthesized nanoparticles has paved the way for plant-mediated biosynthesis of nanoparticles due to their stable and eco-friendly nature in addition to their enhanced biological properties compared to chemically synthesized nanoparticles [2,5,8,9].

*Ipomea* spp. have been in continuous use in nutrition, medicine, rituals, and agriculture [19]. These plant species possess antimicrobial, spasmolytic, analgesic, psychometric, hypotensive, and anticancer activities. The plant is also used to cure inflammation, constipation, kidney ailments, digestive disorders, etc. [19,20]. From the literature, it may be noted that a few studies have been reported on the synthesis of metal nanoparticles from *Ipomea* spp. which have been evaluated for antioxidant and cytotoxic effects [21]; however, to date, no report on the phyto-fabrication of ZnO-NPs from *Ipomea obscura* has been made. Hence, a study on antioxidant, genotoxic, and cytotoxic potentialities of phyto-fabricated zinc oxide nanoparticles (ZnO-NPs) from *I. obscura* aqueous leaf extract was evaluated.

## 2. Materials and Methods

### 2.1. Collection and Phyto-Fabrication of Zinc Oxide Nanoparticles from I. obscura

Healthy leaves of *Ipomea obscura* (L.) Ker Gawl. were collected from Manasagangotri, Mysuru, Karnataka, India and authenticated at the Dept. of Studies in Botany, University of Mysore, Mysuru. Phyto-fabrication of ZnO-NPs from *I. obscura* leaves was carried out according to our previous studies with modifications [8]. In brief, about 25 g of fresh leaves of the plant was collected, washed, and blot dried. The collected sample was then blended in a blender with 250 mL of distilled water and filtered (Whatman No. 1 filter paper). Further, 25 mL of the filtrate was heated to 80 °C (on a magnetic stirrer) and 2.5 g of zinc nitrate hexahydrate was added with constant stirring, and the reaction mixture was stirred until the mixture became paste. The obtained sample was then transferred to a silica furnace and calcinated at 300 °C for 2 h, and the obtained product (ZnO-NPs) was stored in air-tight vials until further use.

### 2.2. Physico-Chemical Characterization of ZnO-NPs

The UV-Vis Spectrophotometer (DU730, Beckman Coulter, Krefeld, Germany) was used to determine the optical density of the phyto-fabricated ZnO-NPs. X-Ray Diffraction (XRD) study was performed out on an X-Ray Diffractometer (Rigaku SmartLab, Tokyo, Japan), and Scherrer’s formula was applied to determine the particle size. The Dynamic Light Scattering (DLS) analysis was also carried out to learn the particle size distribution of ZnO-NPs using the Nanotrack Wave particle size analyzer (Microtrack, PA, USA). The morphology of the nanoparticles was evaluated by Scanning Electron Microscopy (HITACHI-S-3400N, Tokyo, Japan) at 5 kV acceleration. The phyto-fabricated ZnO-NPs were placed on a carbon-coated copper in a tiny amount, allowed to air-dry, and images were taken. The elemental analysis (qualitative as well as quantitative) was carried out by Energy Dispersive Spectroscopy (EDS) (Noran System 7, Thermoscientific, WI, USA). The binding properties of the ZnO-NPs and aqueous extract of *I. obscura* was investigated by FT-IR with a resolution of 4 cm^−1^ between 4000 to 400 cm^−1^ spectral range on a PerkinElmer Spectrum ATR2000 (Singapore).

### 2.3. Evaluation of Phyto-Fabricated ZnO-NPs for Biological Potentialities

#### 2.3.1. 2,2-diphenyl-1-picrylhydrazyl (DPPH) Radical Scavenging Activity

The radical scavenging activity (RSA) of phyto-fabricated ZnO-NPs was performed by following the method of Hemanth Kumar et al. [5]. In brief, 3.5 mL of 0.1 mM DPPH containing 0.2, 0.4, 0.6, 0.8, and 1 mg mL^−1^ concentrations of ZnO-NPs were sonicated before incubating for 30 min at 37 ± 2 °C (under dark conditions) and the absorbance was measured at 517 nm in a spectrophotometer. The percentage of RSA of the nanoparticles was determined.

(1) $$\mathrm{Radical}\;\mathrm{Scavenging}\;\mathrm{Activity}\;\left(\%\right)=\frac{A-\mathrm{bsorbance}\;\mathrm{of}\;\mathrm{Control}\;-\;\mathrm{Absorbance}\;\mathrm{of}\;\mathrm{Test}\;\mathrm{sample}}{A-\mathrm{bsorbance}\;\mathrm{of}\;\mathrm{Control}\;}\;\times \;100$$

#### 2.3.2. Genotoxic Analysis of Phyto-Fabricated ZnO-NPs by the Allium cepa Method

The genotoxicity of ZnO-NPs was determined in the root tips of healthy onion bulbs [5]. Onion bulbs with fresh roots (2–3 cm long) grown on glass vials were transferred to glass vials containing phyto-fabricated ZnO-NPs (0.2, 0.4, 0.6, 0.8, and 1 mg mL^−1^) and were subjected to incubation for 24 h at room temperature. The ZnO-NPs treated onion root tips were watchfully cut out and fixed in Carnoy’s Fixative II (for 24 h) and later relocated in 70% ethanol. Later, the root tips were taken and squashed, and the cells were observed for cell division and, also, to record the mitotic index. Sterile Distilled Water (SDW) and Methotrexate treated root tips were designated as the negative and positive control, respectively. A total of ten microscopic fields per root sample were observed with four roots per treatment.

(2) $$\mathrm{Mitotic}\;\mathrm{Index}\;\left(\%\right)=\frac{\mathrm{Number}\;\mathrm{of}\;\mathrm{dividing}\;\mathrm{cells}}{\mathrm{Total}\;\mathrm{number}\;\mathrm{of}\;\mathrm{cells}}\times 100$$

#### 2.3.3. Cytotoxic Analysis of Phyto-Fabricated ZnO-NPs

##### 3-(4,5-dimethylthiazol-2-yl)-2,5-diphenyl tetrazolium bromide (MTT) Assay

MTT assay was carried out to identify the anticancer properties of phyto-fabricated ZnO-NPs according to the method of Selvakumaran et al. [22]. Cell lines (HT-29) (procured from NCCS, Pune, India) of 80% confluent cells were trypsinized. Each well was seeded with 100 µL of cell suspension (5 × 10^5^) in Dulbecco’s modified Eagle’s medium (DMEM) and further incubated for 24 h at 37 °C (humidified atmosphere containing 5% CO_2_). After incubation, the media were discarded, and each well was loaded with 150 µL of fresh DMEM media containing ZnO-NPs (0.2, 0.4, 0.6, 0.8, and 1 mg) with fetal bovine serum (10%) and incubated for 24 h. The incubated samples were aspirated and 100 µL of MTT solution (0.5 mg mL^−1^ in 1× PBS filtered through 0.2 µM filter) was added and subjected to incubation for 4 h. From the incubated samples, MTT reagent was removed and DMSO (100 µL) was added to solubilize formazan rapidly. SDW and colchicine treated cells were designated as a negative and positive control, respectively. Each sample was subjected to UV-Vis spectroscopy at 570 nm, and the percentage of cell viability was calculated.

(3) $$\mathrm{Cell}\;\mathrm{Viability}\;\left(\%\right)=\frac{\mathrm{Absorbance}\;\mathrm{of}\;\mathrm{sample}}{\mathrm{Absorbance}\;\mathrm{of}\;\mathrm{control}}\times \;100$$

##### Cell Cycle Analysis

About 3 mL well^−1^ of HT-29 cell suspension (5 × 10^5^) was loaded into each well of a 6-well plate and subjected to incubation at 37 °C for 24 h (5% CO_2_). After incubation, the growth media were discarded by aspiration and reloaded with 1 mL of phyto-fabricated ZnO-NPs (1 mg) and colchicine (320 µg) before subjecting to incubation in humified conditions. The treated cells were detached by trypsinization and subjected to centrifugation for 10 min at 2000 rpm and repeatedly washed with PBS. Later, the cell pellet was fixed with ethanol (700 µL) and further incubated (for 1 h at −20 °C). Subsequently, the cells were washed twice with PBS (ice cold) by cold centrifugation for 10 min at 4000 rpm. The resultant cell pellet was resuspended in PBS (1 mL) containing propidium iodide (50 mg mL^−1^), RNase A (50 mg mL^−1^), and Triton X-100 (0.1%) and incubated for 30 min under dark conditions before analyzing using a flow cytometer (Cell Lab Quanta™, SC, Beckman Coulter, CA, USA).

##### Annexin V-FITC Staining Assay

The phyto-fabricated ZnO-NPs treated (1 mg) and respective control cells, as mentioned above, were subjected to AnnexinV-FITC staining by using Annexin V-FITC Apoptosis Detection Kit (Invitrogen, USA) following the manufacturer’s protocol. In brief, the ZnO-NP treated and untreated cells were resuspended in binding buffer (100 µL) containing PI (10 µL) and subjected to incubation for 5 min (dark conditions). The stained cell samples were further subjected to analysis by a flow cytometer (Cell Lab Quanta™, SC, Beckman Coulter, CA, USA).

##### Analysis of Cell Morphology

Morphological changes in HT-29 cells upon treatment with phyto-fabricated ZnO-NPs were performed on a 6 well-plate. Each well was seeded with 1 mL of DMEM media containing HT-29 cells (1 × 10^5^ cells mL^−1^) and incubated for 24 h. The HT-29 cell suspension was discarded after incubation by aspiration and loaded with 1 mL of fresh DMEM media containing ZnO-NPs (1 mg mL^−1^). The treated samples were incubated at room temperature for 24 h aseptically. After incubation, the cellular morphology changes were observed using phase-contrast inverted microscopy at 20X magnification (Zeiss Axio Vert. A1, Jena, Germany). 

### 2.4. Statistical Analysis 

The experimental studies were carried out with four replicates for each experiment. They were statistically analyzed by subjecting to arcsine transformation and analysis of variance (ANOVA) using SPSS, version 17 (SPSS Inc., Chicago, IL, USA). The significant differences between the treatment mean values were determined by the Honestly Significant Difference (HSD) obtained by Tukey’s test at *p* ≤ 0.05 levels.

## 3. Results and Discussion 

### 3.1. Physico-Chemical Characterization of Phyto-Fabricated ZnO-NPs

**Figure 1:**
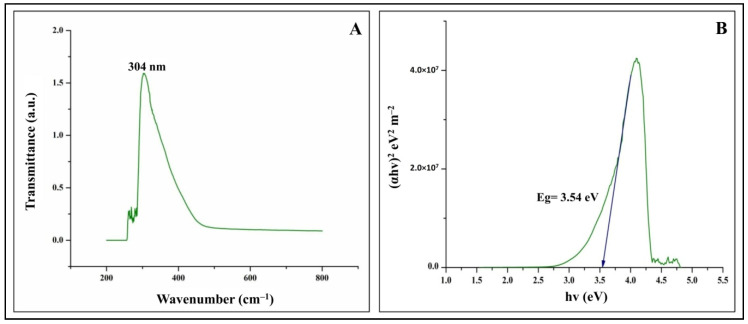
UV-visible absorption spectroscopy (**A**) and band gap energy (**B**) of phyto-fabricated zinc oxide nanoparticles (ZnO-NPs) from *I. obscura* aqueous leaf extract.

**Figure 2:**
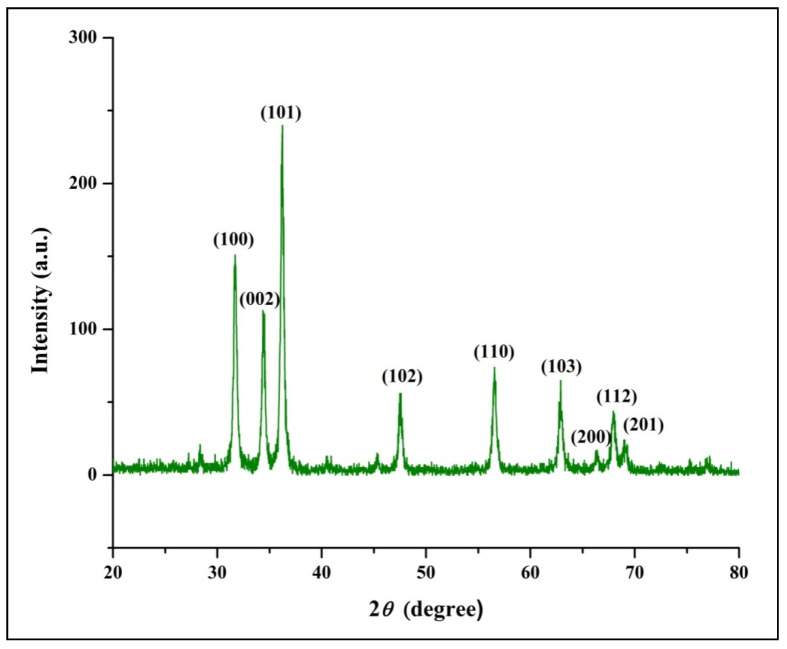
X-Ray Diffraction analysis of phyto-fabricated ZnO-NPs from *I. obscura* aqueous leaf extract.

**Figure 3:**
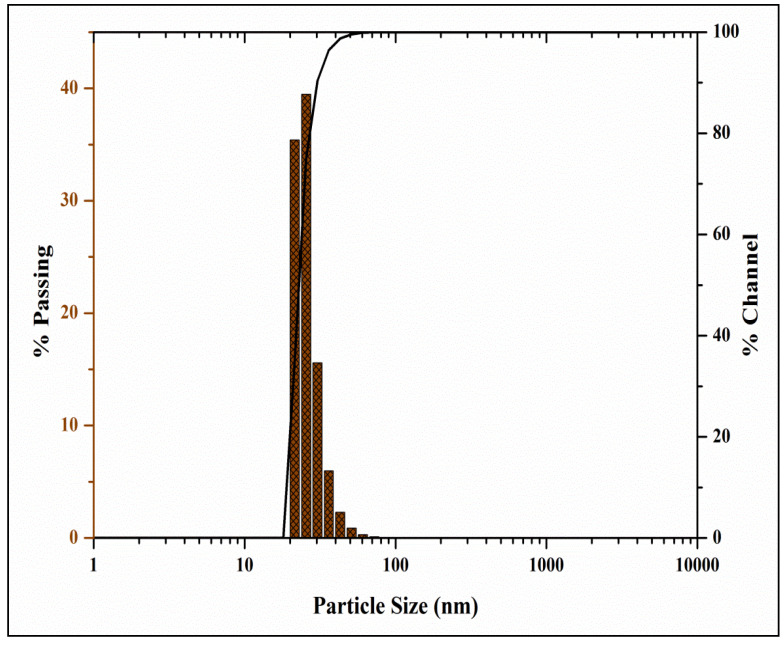
Dynamic Light Scattering Analysis of phyto-fabricated ZnO-NPs from *I. obscura* aqueous leaf extract.

**Figure 4:**
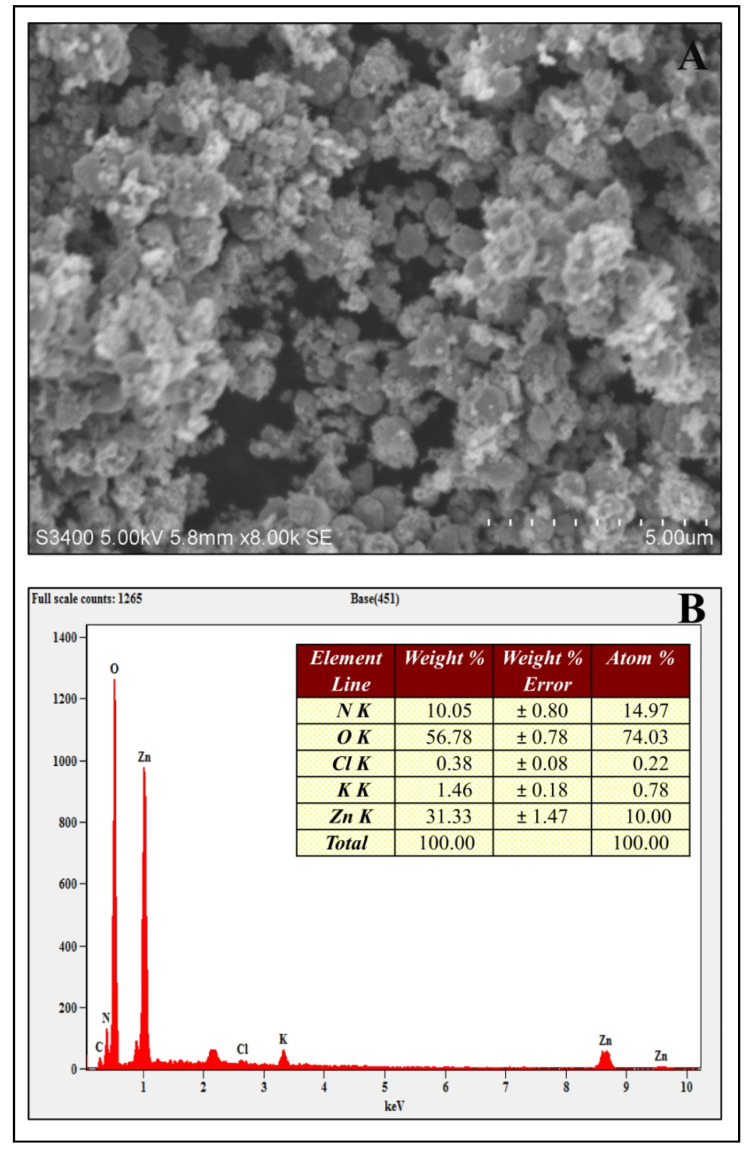
Scanning Electron Micrograph (**A**) and Energy Dispersive Spectra (**B**) of phyto-fabricated ZnO-NPs.

**Figure 5:**
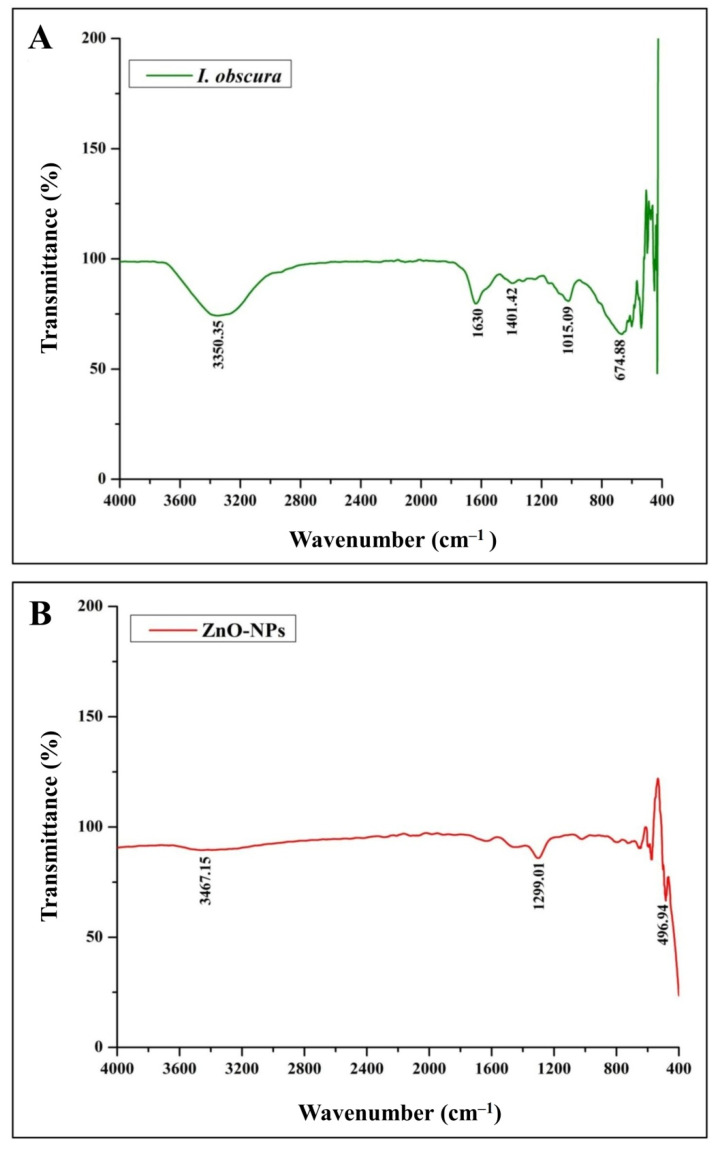
FT-IR analysis of *I. obscura* aqueous leaf extract (**A**) and its phyto-fabricated ZnO-NPs (**B**).

The phyto-fabricated ZnO-NPs offered an absorption peak of 304 nm (Figure 1A) with an energy band gap of 3.54 eV (Figure 1B). The spectral analysis results agreed with the results of many other studies in which the absorption peak and bandgap energy of phyto-fabricated ZnO nanoparticles were between 280 to 400 nm and 3.2 eV to 3.5 eV, respectively, which is crucial for their applications in biological and pharmaceutical fields [23]. The XRD analysis of nanoparticles revealed stiff narrow diffraction peaks, thereby confirming that the particles were without any impurities (Figure 2). The crystalline size of the nanoparticles was ~24.26 nm upon calculation through Scherrer’s formula based on the full-width half-maximum (FWHM) of the stiff narrow peaks (Table S1 in Supplementary Materials) and well-matched to JCPDS No. 65-3411. The results of XRD analysis conformed with the findings of Hemanth Kumar et al. [13] and Lakshmeesha et al. [24], wherein plant-based synthesis of ZnO-NPs were of 15–60 nm in size, which was calculated based on FWHM of the peaks. The results of the DLS analysis revealed that the phyto-fabricated ZnO-NPs were of an average diameter of 22.78 nm (Figure 3) in the aqueous colloidal solution, which is in agreement with the results obtained from the XRD analysis. The morphology of phyto-fabricated ZnO-NPs visualized under SEM showed agglomeration of nanoparticles (Figure 4A). Further insight on the quantitative elemental analysis of ZnO-NPs through EDS revealed that the particles were of 88.11% purity (Figure 4B). Correspondingly, researchers have reported that the biosynthesis of ZnO-NPs from the extracts of plant materials exemplify better purity, which has been confirmed by XRD, SEM, and EDS analysis [6,9,14,16]. The FT-IR spectroscopic analysis of ZnO-NPs and aqueous plant extract helped to recognize the functional groups that were responsible for the phyto-fabrication of the nanoparticles during the reduction and stabilization. The spectrum band at 496.94 cm^−1^ in ZnO-NPs (Figure 4A) confirmed the formation of the metal oxide bond, while the non-appearance of the peaks around 3350 cm^−1^ (Figure 5) indicates the particles were moisture-free (Table S2 in Supplementary Materials). It may be noted through the FT-IR spectrum of ZnO-NPs that functional groups present in plant extract had complexed well to form a metal oxide as indicated by the absence of major peaks between 600 and 400 cm^−1^ [2,5,6]. The results affirm that during the phyto-fabrication of ZnO-NPs, the metabolites in plant extract act as capping/stabilizing agents to form a metal oxide, which is also in agreement with the conclusions of other researchers [6,9,25].

### 3.2. Evaluation of Phyto-Fabricated ZnO-NPs for Biological Potentialities

#### 3.2.1. DPPH Radical Scavenging Activity

**Figure 6:**
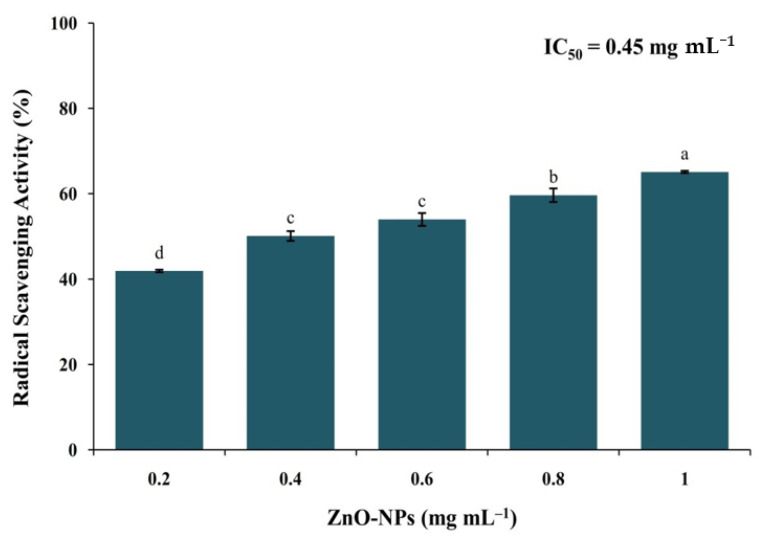
DPPH radical scavenging activity of phyto-fabricated ZnO-NPs from *I. obscura* aqueous leaf extract. Each value is the mean for three replicates (n = 3), and bars sharing the same letters are not significantly different (*p* ≤ 0.05) according to Tukey’s Honestly Significant Difference (HSD). The vertical bar indicates the standard error.

The percent RSA of ZnO-NPs from *I. obscura* increases with an increase in the particles’ dose with a half-maximal inhibitory concentration (IC50) of 0.45 mg mL^−1^ (Figure 6). In comparison, ascorbic acid (positive control) possessed 75% inhibition at 50 µg mL^−1^. It has been well-documented that the hydroxyl free radicals result in DNA damage and lipid peroxidation, which is directly correlated to cancer-related complications [5]. The results obtained from the study are in corroboration with the findings of other researchers in which ZnO-NPs synthesized using biological route possessed effective antioxidant activity [5,26]. The antioxidant nature of the green synthesized ZnO-NPs may be correlated to the secondary metabolites that act as agents of capping/reducing/stabilizing during nanoparticle synthesis [10,21].

#### 3.2.2. Genotoxic Analysis of Phyto-Fabricated ZnO-NPs by the Allium cepa Method

**Figure 7:**
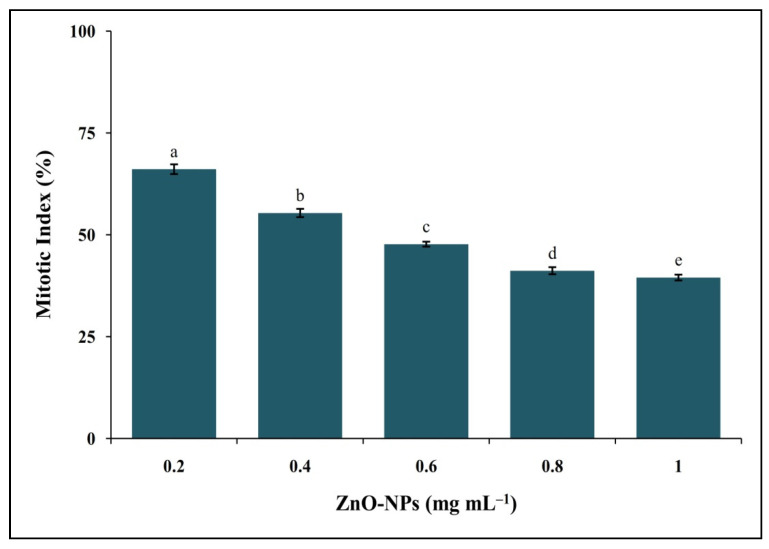
Genotoxic potential of phyto-fabricated ZnO-NPs from *I. obscura* aqueous leaf extract. Each value is the mean for three replicates (n = 3) and bars sharing the same letters are not significantly different (*p* ≤ 0.05) according to Tukey’s HSD. The vertical bar indicates the standard error.

**Figure 8:**
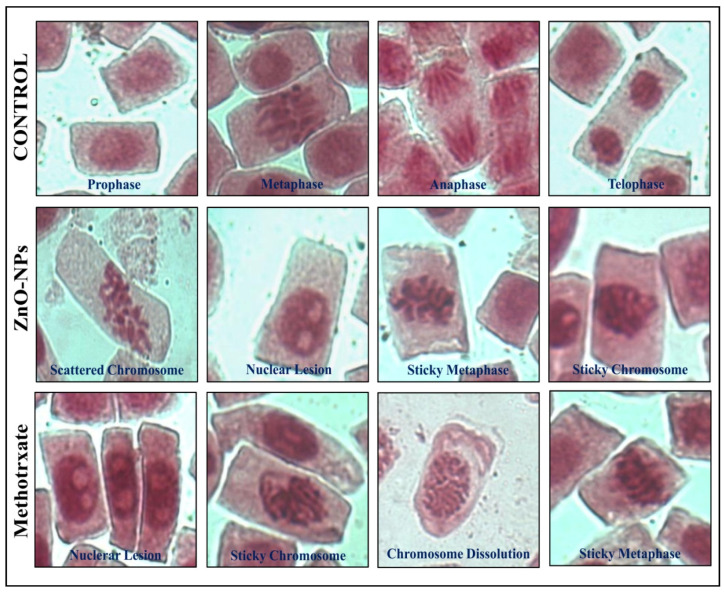
Representative images of chromosomal aberrations observed in onion root meristem cells upon treatment with ZnO-NPs.

*Allium cepa* L. is considered a bioindicator species for genotoxicity assays by the United States Environmental Protection Agency (USEPA) and the United Nations Environmental Programme (UNEP) [23]. The antimitotic potentiality of phyto-fabricated ZnO-NPs was analyzed using *A. cepa* root tips, and the results of the study are presented in Figure 7. The results of the study indicated that the mitotic index decreased in a dose-dependent manner up to 39.49% (Table S3 in Supplementary Materials), thereby indicating a potent inhibitory effect against the cell division. The microscopic studies of onion root tips exposed to ZnO-NPs showed chromosomal abnormalities in more cells than untreated control root tips, where typical cell divisions were observed (Figure 8). The inhibition of cell division upon exposure to ZnO-NPs affirms the genotoxic nature of the nanoparticles (cell arrest in mitosis eventually leading to apoptosis) and conforms with the findings of other researchers wherein ZnO-NPs synthesized from plant extracts offered effective antimitotic properties [13,23,27]. Further, in our previous studies, we had evaluated the effect of commercially available zinc oxide for their genotoxicity and found that there was no significant genotoxic effect of the treatment, but normal cell divisions were noticed even after treating at 2.5 mg mL^−1^ concentration [12]. It has been widely reported that ZnO-NPs synthesized using plant extracts with genotoxic nature will also have potential anticancer properties [28,29].

#### 3.2.3. Cytotoxic Analysis of Phyto-Fabricated ZnO-NPs

##### MTT Assay

**Figure 9:**
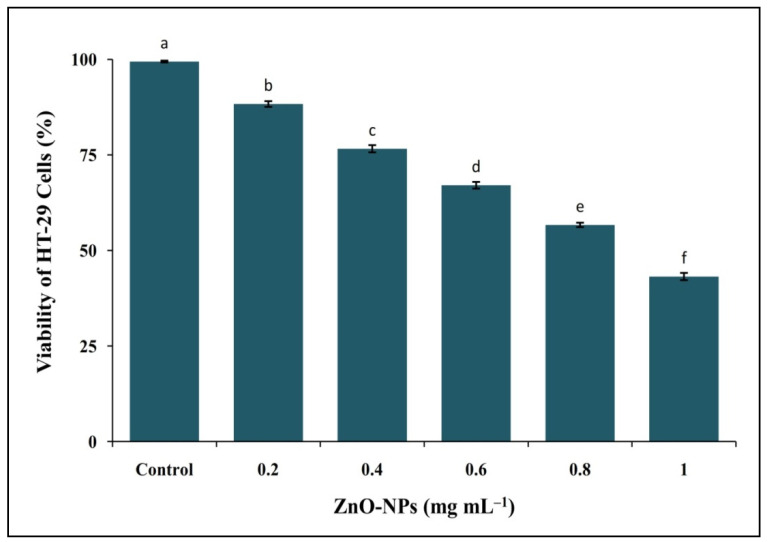
In vitro cytotoxicity study of phyto-fabricated ZnO-NPs from *I. obscura* aqueous leaf extract against HT-29 cell lines by MTT assay. Each value is the mean for three replicates (n = 3) and bars sharing the same letters are not significantly different (*p* ≤ 0.05) according to Tukey’s HSD. The vertical bar indicates the standard error.

The cytotoxic effect of ZnO-NPs from *I. obscura* was evaluated by MTT assay against HT-29 cells. The assay studied is considered to be one of the most reliable methods to primarily assess the effect of compounds on cell lines [30]. The phyto-fabricated ZnO-NPs gave dose-dependent cytotoxicity wherein the cell viability decreased from 88.32% to 43.17% (0.2 to 1 mg mL^−1^) with an IC50 value of 0.91 mg mL^−1^. The standard drug colchicine offered around 70% cytotoxicity at 100 µg mL^−1^. A significant rise in the percent cell inhibition was observed in a dose-dependent manner compared to control (Figure 9). Likewise, biosynthesized ZnO-NPs inhibited human breast cancer cell lines (MDA-MB 231 and MCF-7) evaluated through MTT assay and correlated to its antioxidant potentiality [31].

##### Cell Cycle Analysis

**Figure 10:**
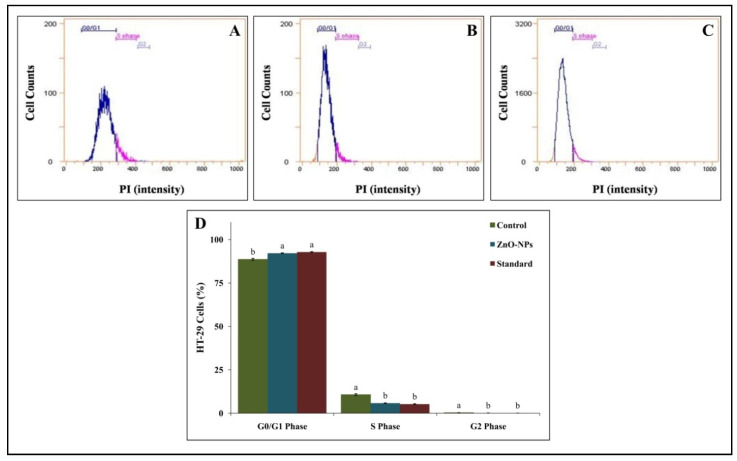
Representative graph of flow cytometry of HT-29 cell cycle analysis. (**A**): Control; (**B**): ZnO-NPs; (**C**): Colchicine; (**D**): Percentage of HT-29 cells. Each value is the mean of three replicates (n = 3) and bars sharing the same letters are not significantly different (*p* ≤ 0.05) according to Tukey’s HSD. The vertical bar indicates the standard error.

The cell cycle includes G1, S, G2, and mitosis (M); in brief, in the G1 phase, RNA and proteins are produced for the DNA replication, the S phase includes DNA synthesis, and in the G2 phase, new proteins are synthesized for cell division, whereas in the M stage, nuclear and cytoplasmic divisions take place [32]. It is well noted that anticancer agents can arrest the cell cycle before inducing apoptosis [33]. Similarly, ZnO-NP treatment was able to arrest the cell cycle at the G0/ G1 phase along with a significant reduction in the accumulation of cells at the S phase after 24 h of treatment in the present study (Figure 10). A total of 92.14% (G0/G1 phase), 5.82% (S phase), and 0.12% (G2 phase) HT-29 cells were observed after 24 h treatment with ZnO-NPs (Figure 10B). Further, it was also noted that the results on cell division upon treatment with ZnO-NPs were comparable to the effects of the standard drug (Figure 10D). In accordance with the results, Kaminskyy et al. [34] reported that molecules/drugs have the ability to arrest the cell cycle in the G2/ M or S phase, which was directly correlated to their sensitivity of the cell lines toward the anticancer agents.

##### Annexin V-FITC Staining Assay

**Figure 11:**
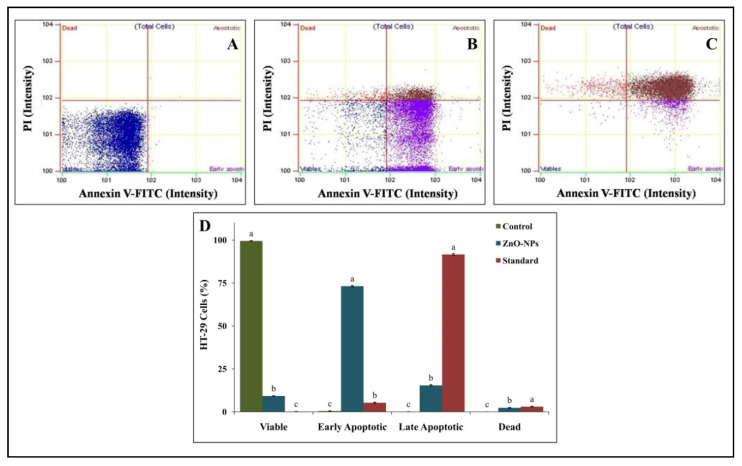
Representative graph of flow cytometry analysis of HT-29 cells with Annexin-V fluorescein isothiocyanate (FITC). (**A**): Control; (**B**): ZnO-NPs; (**C**): Colchicine; (**D**): Percentage of HT-29 cells. Each value is the mean of three replicates (n = 3) and bars sharing the same letters are not significantly different (*p* ≤ 0.05) according to Tukey’s HSD. The vertical bar indicates the standard error.

The biochemical hallmarks of cancer include cleavage of intracellular substrates, DNA fragmentation, and phosphatidylserine (PS), which is actively localized on the inner leaflet of the plasma membrane in healthy cells [35]. The flipping of its distribution is generally accepted as one of the apoptotic biomarkers [36]. The loss of PS asymmetry can be detected with the staining of Annexin V, which specifically binds PS; the latter can be detected by flow cytometry when fluorescently conjugated [37]. Likewise, Annexin V/ PI double staining was used to determine the effect of ZnO-NPs upon treatment of HT-29 cells to detect the stages of apoptosis (Figure 11). ZnO-NPs induced early apoptotic (73.14%) at 24 h, whereas upon colchicine treatment, there was abigger increase in late apoptotic cells (91.61%) than in early apoptotic cells (5.24%). The results of the study affirm that the ZnO-NPs may have generated ROS and oxidative stress, which leads to apoptosis in the HT-29 cell line. Consistent with our results, ZnO-NPs were able to induce cytotoxicity in LTEP-a-2 cells [38]. In agreement with the results of the present study, Bai et al. [39] reported the induction of significant cytotoxicity in human ovarian cells upon treatment with ZnO-NPS and correlated the cytotoxic effect of the nanoparticles to the generation of ROS and oxidative stress.

##### Morphological Evaluation HT-29 Cells by Phase-Contrast Microscopy

**Figure 12:**
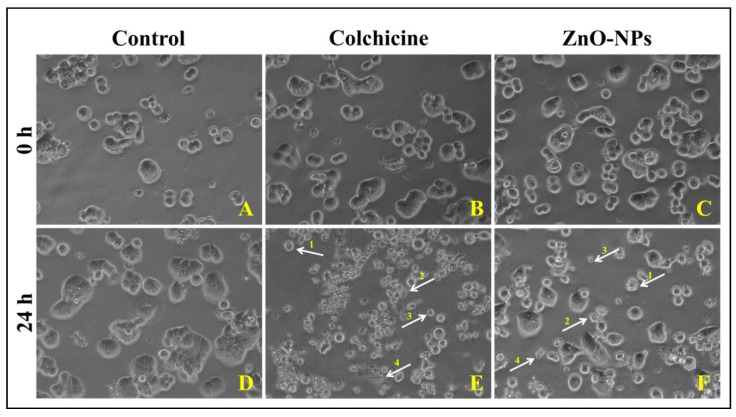
Representative phase-contrast inverted microscopic images showing the morphological changes observed in HT-29 cells upon treatment with phyto-fabricated ZnO-NPs. (**A**,**D**): Control; (**B**,**E**): ZnO-NPs treated; (**C**,**F**): Colchicine treated; 1: Membrane blebbing; 2: Nuclear fragmentation; 3: Cell shrinkage; 4: Apoptotic bodies.

The morphological changes within the cells were evaluated to authenticate the results obtained from the cytotoxic studies. It was observed that ZnO-NPs and colchicine treated HT-29 cells showed morphological changes such as blebbing in the cell membrane, shrinkage of cells, the formation of apoptotic bodies, nuclear fragmentation, and loss of membrane stability (Figure 12E,F). In contrast, no morphological changes were observed in control HT-29 cells even after 24 h incubation (Figure 12C). Similar to the observations of the present study, Vijaykumar et al. [40] also observed morphological changes in A549 cells upon treatment with ZnO-NPs green synthesized using *Laurus nobilis*. These changes in the morphology in cell lines upon treatment with ZnO-NPs are correlated to the intracellular ROS generation, dysfunction of mitochondria, and leakage of plasma membrane leading to cell death [41]. From the studies, we presume that the ZnO-NP exposure to HT-29 cells will induce the overproduction of ROS, which leads to the over expression of the caspase-12 protein in HT-29 cells, thus triggering the ER stress resulting in HT-29 cell damage and finally inducing apoptosis/necrosis in HT-29 cells [38]. The results of the study confirm that the phyto-fabricated ZnO-NPs possess effective cytotoxic properties against HT-29 cells.

## 4. Conclusions

In conclusion, the process of phyto-fabrication of ZnO-NPs from *I. obscura* leaf extract was carried out for the first time in the present study. The physico-chemical characterization of the nanoparticles possessed bandgap energy of 3.54 eV, ~24.26 nm size with 88.11% purity. The obtained nanoparticles were free from moisture, as evidenced through FT-IR analysis. The antioxidant study revealed dose-dependent RSA with the particles showing IC50 of 0.45 mg mL^−1^. Further, the phyto-fabricated ZnO-NPs showed genotoxicity and cytotoxicity against *A. cepa* meristem and HT-29 cells, respectively, which were comparable results to that of standard drugs used. The results affirm that the phyto-fabricated ZnO-NPs could be a potent substitute for the synthetic drug used presently for cytotoxicity.

## Data Availability

The data presented in this study are available in this manuscript.

## Supplementary Materials

The following are available online, Table S1: Estimated crystallite size of phyto-fabricated ZnO-NPs from *I. obscura* leaf extract; Table S2: FT-IR spectra with possible assignments of phyto-fabricated ZnO-NPs and *I. obscura* leaf extract; Table S3: Genotoxicity of phyto-fabricated ZnO-NPs from *I. obscura* leaf extract.

## Sample Availability

Samples of the compounds are available from the authors.
