# Supplementary material for: Genotoxic and Cytotoxic Properties of Zinc Oxide Nanoparticles Phyto-Fabricated from the Obscure Morning Glory Plant Ipomoea obscura (L.) Ker Gawl
Source: Molecules. 2021 Feb 8;26(4):891. doi: 10.3390/molecules26040891 (PMC7915295; doi:10.3390/molecules26040891)
Supplement: Supplementary file 1 [file molecules-26-00891-s001.pdf]

Supplementary Table 1: Estimated crystallite size of phyto-fabricated ZnO-NPs from *I. obscura* leaf extract

	**2θ (deg.)**	**(*hkl*)**	**FWHM**	**Crystalline Size (nm)**	**Average Crystalline Size (nm)**
**Biosynthesized ZnO-NPs**	31.65	(100)	0.3459	24.95	24.26
	34.47	(002)	0.3527	24.65	
	36.17	(101)	0.366	23.87	
	47.43	(102)	0.397	22.85	
	56.48	(110)	0.4201	22.44	
	62.83	(103)	0.3475	28.00	
	66.35	(200)	0.3838	25.85	
	67.86	(112)	0.3745	26.72	
	68.98	(201)	0.5302	19.00	

Supplementary Table 2: FT-IR spectra with possible assignments of phyto-fabricated ZnO-NPs and *I. obscura* leaf extract

**Frequency (cm^{-1})**	**Possible assignment**	**Functional Group**
***Ipomoea obscura* extract**		
3350.35	O-H stretch	Alcohol/ Phenol
1630	N-H bend	Primary amines
1401.42	C-C bend	Aromatic carbon
1015.09	C-N bend	Aliphatic amine
674.88	- C=C – H stretch	Alkyne
**Phyto-fabricated ZnO-NPs**		
496.94	Zn-O	Zinc oxide

Supplementary Table 3: Genotoxicity of phyto-fabricated ZnO-NPs from *I. obscura* leaf extract

Treatments ($mg\ mL^{-1}$)	Mitotic Index (%)	Dividing Cells (%)	Mitotic Inhibition (%)
0.2	66.08 ± 1.19[a]	44.66 ± 0.99[a]	55.30 ± 1.02[e]
0.4	55.33 ± 1.02[b]	42.70 ± 1.44[a]	57.29 ± 1.44[e]
0.6	47.68 ± 0.60[c]	34.96 ± 0.93[b]	65.03 ± 0.93[d]
0.8	41.17 ± 0.86[d]	30.83 ± 0.89[c]	69.17 ± 0.89[c]
1.0	39.49 ± 0.71[d]	24.68 ± 1.18[d]	75.15 ± 1.02[b]
Methotrexate	15.89 ± 0.45[e]	18.43 ± 0.56[e]	81.68 ± 0.80[a]

Values are means of three independent replicates (n = 3) and ± indicates standard error. Means followed by the same letter(s) within the same column are not significantly ($p \leq 0.05$) different according to Tukey's HSD.
